# Integrated Diagnostics for Atrial Fibrillation Recurrence: Exploratory Results from the PLACEBO Trial

**DOI:** 10.3390/diagnostics15091105

**Published:** 2025-04-27

**Authors:** Aristi Boulmpou, Theodoros Moysiadis, Georgios Zormpas, Eleftherios Teperikidis, Konstantina Tsioni, Maria Toumpourleka, Maria Zidrou, Georgios Giannakoulas, Vassilios Vassilikos, Christodoulos Papadopoulos

**Affiliations:** 1 Third Department of Cardiology, Ippokratio General Hospital, Aristotle University of Thessaloniki, 54642 Thessaloniki, Greece; 2 Department of Computer Science, School of Sciences and Engineering, University of Nicosia, 2417 Nicosia, Cyprus; 3 Second Department of Cardiology, Ippokratio General Hospital, Aristotle University of Thessaloniki, 54642 Thessaloniki, Greece; 4 Biopathology Laboratory, Ippokratio General Hospital, 54642 Thessaloniki, Greece; 5 First Department of Cardiology, AHEPA University Hospital, Aristotle University of Thessaloniki, 54636 Thessaloniki, Greece

**Keywords:** paroxysmal atrial fibrillation, CPET, echocardiography, biomarkers, risk stratification

## Abstract

**Background**: Atrial fibrillation is a prevalent arrhythmia with significant morbidity and recurrence challenges. Paroxysmal atrial fibrillation (PAF) is characterized by episodic occurrences and unpredictable recurrences; therefore, it demands innovative diagnostic approaches to predict relapses and guide management. **Objectives**: This pilot, exploratory study evaluates the feasibility and prognostic value of integrating cardiopulmonary exercise testing (CPET), echocardiographic indices, and plasma biomarkers for predicting PAF recurrence. **Methods**: The PLACEBO trial is a single-center, prospective observational study of 73 adults with PAF in sinus rhythm at baseline. Comprehensive assessments included CPET, transthoracic echocardiography, 24 h electrocardiographic Holter monitoring with heart rate variability (HRV) metrics, and plasma biomarkers, such as galectin-3 (GAL3). Recurrence was defined as any documented AF episode lasting ≥30 s within 12 months of follow-up. **Results**: Binary logistic regression revealed that the standard deviation of RR intervals (SDRR) and GAL3 were significant predictors of recurrence. Particularly, higher SDRR [odds ratio (OR): 1.061, *p* = 0.021] and GAL3 > 10.95 ng/mL (OR: 5.206, *p* = 0.006) were associated with recurrence. Moreover, lower right ventricular fractional area change (RV FAC) exhibited a marginally significant association with recurrence (OR: 0.927, *p* = 0.062). CPET parameters demonstrated limited prognostic value in this cohort. **Conclusion**: This pilot study demonstrates that integrating novel echocardiographic indices, biomarkers, and HRV metrics is feasible and may provide valuable prognostic insights for PAF recurrence. Larger multicenter studies are needed to validate these findings and optimize personalized risk stratification strategies.

## 1. Introduction

Atrial fibrillation (AF) is the most common cardiac arrhythmia worldwide, contributing significantly to morbidity and mortality [1]. AF leads to rising healthcare costs, whilst posing a major impact on the health-related quality of life of the affected subjects [2,3]. AF presents with various subtypes, including paroxysmal, persistent, and permanent forms, each characterized by unique clinical patterns and challenges in management [4]. Specifically, paroxysmal atrial fibrillation (PAF) is characterized by its episodic nature, unpredictable recurrences, and variable clinical outcomes; even though PAF seems to confer a more favorable prognosis compared to other forms of AF, the effective management and refinement of the prognosis of the disease is crucial [5,6]. Despite advancements in AF management, predicting recurrence remains a major clinical challenge, necessitating a comprehensive approach that integrates advanced diagnostic modalities.

Cardiopulmonary exercise testing (CPET) is a valuable tool for the comprehensive assessment of the function of cardiovascular, respiratory, and musculoskeletal systems, commonly used in clinical practice with the aim of evaluating the multiorgan response to exercise [7]. The role of CPET in the assessment of patients with AF has already been established, while the exact relationship between cardiorespiratory fitness and AF course prognosis is not yet fully identified [8]. In the same line, a series of measurements obtained from transthoracic echocardiography (TTE) is crucial for the evaluation of patients with AF, as they aid in the assessment of structural and functional cardiac changes that influence patient outcomes; novel echocardiographic indices such as right ventricular fractional area change (RV FAC) and left atrial (LA) strain offer detailed cardiac function assessments in patients with AF [9,10,11]. Finally, plasma biomarkers, such as galectin-3 (GAL3) and homocysteine (Hcy), are established tools for evaluating cardiovascular health, as they are directly linked with fibrosis and inflammation [12,13]. The combined prognostic value of CPET, TTE measurements, and biomarker evaluation in PAF is not yet well understood.

Heart rate variability (HRV) metrics, such as the standard deviation of R-R intervals (SDRR) and the standard deviation of normal-to-normal R-R intervals (SDNN), offer valuable insights into the autonomic regulation of the heart and have been increasingly explored in AF [14]. While reduced HRV is well established as a marker of poor prognosis in AF, emerging evidence suggests that increased HRV may also play a role in AF recurrence, possibly through heightened vagal tone, which contributes to arrhythmogenesis [15]. Understanding the complex relationship between HRV and AF may help refine risk stratification and management strategies.

Despite advancements in risk stratification for PAF, the existing models often focus on isolated parameters, such as structural echocardiographic indices or single biomarkers. This fragmented approach may overlook the interplay between hemodynamic, biochemical, and structural changes. Our study bridges this gap by integrating CPET, novel echocardiographic indices, and plasma biomarkers, aiming to create a unified predictive model for PAF recurrence [16,17]. In this context, the PLACEBO exploratory trial aims to integrate the aforementioned diagnostic modalities to enhance our understanding of PAF recurrence. This manuscript presents the exploratory results of the study, as well as their implications for clinical practice.

## 2. Materials and Methods

### 2.1. Study Design and Setting

The PLACEBO (ParoxysmaL Atrial fibrillation prognosis based on Cardiopulmonary Exercise test data and novel echocardiographic and plasma BiOchemical indices) trial is a prospective, exploratory, single-center, observational cohort study conducted at Ippokratio General Hospital in Thessaloniki, Greece. The protocol of the trial is registered at ClinicalTrials.gov (Identifier: NCT05246423) and was reviewed and approved by the Institutional Review Board of Ippokratio General Hospital of Thessaloniki, as well as by the Aristotle University of Thessaloniki Ethics Committee. The study was conducted according to the latest version of the Declaration of Helsinki (2013), and all participants provided informed consent prior to study enrollment. Adults diagnosed with PAF and in sinus rhythm at baseline were included. The study was designed to comprehensively evaluate novel diagnostic and prognostic parameters for PAF recurrence through an integrative approach, encompassing advanced cardiac imaging, biomarker analysis, HRV metrics, and functional assessment. The rationale, design, and protocol of the study were previously published [18].

### 2.2. Study Population

The study participants were recruited from the cardiology outpatient clinic and inpatient referrals at Ippokratio General Hospital of Thessaloniki, ensuring a diverse yet representative cohort of patients with PAF.

The inclusion criteria were (a) age ≥ 18 years, (b) diagnosis of PAF, (c) sinus rhythm at the time of baseline evaluation, (d) ability to provide written informed consent, (e) ability to undergo CPET, and (f) ability to comply with this study’s follow-up schedule.

The exclusion criteria were (a) structural cardiomyopathy, (b) congenital heart disease, (c) permanent AF, (d) recent acute coronary syndrome (within the last month), (e) heart failure, (f) end-stage renal disease, (g) autoimmune disease, (h) active malignancy, (i) uncontrolled thyroid disease, (j) recent surgery (within the last 2 months), (k) poor echocardiographic windows, (l) uncontrolled hypertension, (m) pregnancy, and (n) inability to comply with the study protocol or to provide informed consent.

**Figure 1:**
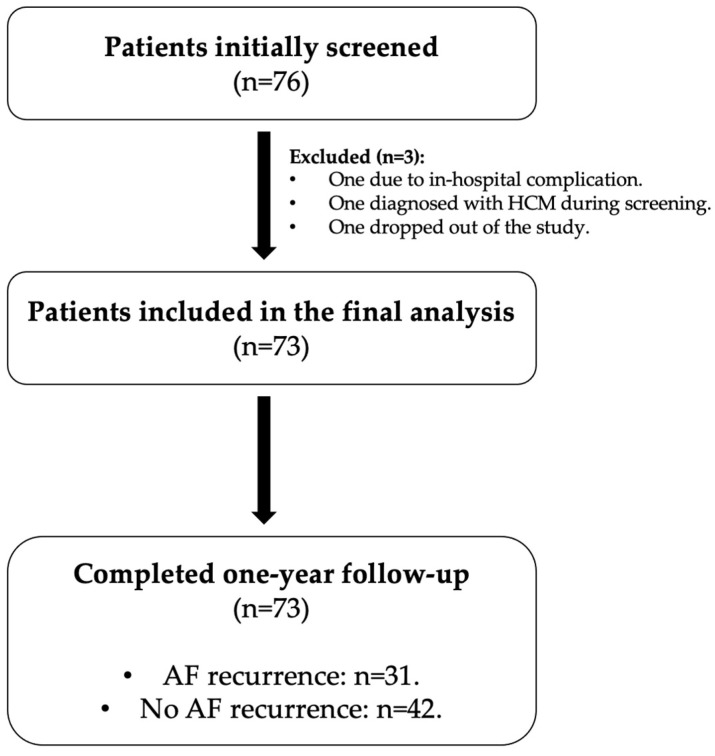
Study flowchart of patient enrollment and follow-up. Abbreviations: HCM, hypertrophic cardiomyopathy; AF, atrial fibrillation.

A total of 76 patients were initially screened for participation. Three patients were excluded, including one due to an in-hospital complication, one due to a new diagnosis of hypertrophic cardiomyopathy during screening, and one who failed to complete the follow-up requirements. Thus, 73 patients were included in the final analysis and were followed for one year for AF recurrence. The study flowchart is presented in Figure 1.

### 2.3. Data Collection

#### 2.3.1. Baseline

At baseline, the participants underwent comprehensive evaluations, including the following:

1. Demographics and medical history: Demographic data [e.g., age, sex, body mass index (BMI)] and detailed clinical history, including cardiovascular risk factors, comorbidities, prior PAF episodes, and their characteristics, were recorded.
2. CPET: CPET was performed on a cycle ergometer with continuous 12-lead ECG monitoring and respiratory gas analysis. The key parameters obtained included peak oxygen uptake (peak VO_2_), minute ventilation/carbon dioxide production (VE/VCO_2_), and other exercise indices with established prognostic value in cardiovascular conditions.
3. Transthoracic echocardiography: A comprehensive echocardiographic assessment was conducted under stable hemodynamic conditions. The parameters included left ventricular (LV) ejection fraction (LVEF), global longitudinal strain (GLS), LV diastolic indices, and LA dimensions, volume, and strain. Right ventricular parameters, such as RV FAC and free wall strain, were also evaluated. All echocardiographic examinations were performed by a single experienced cardiologist to ensure consistency. For the echocardiographic measurements, the GE Vivid E95 ultrasound system (GE Healthcare, Chicago, IL, USA) was used. The 2D measurements were performed offline using the commercially available EchoPAC GE software package, version PC (GE Healthcare, Chicago, IL, USA). All measurements adhered to the European Association of Cardiovascular Imaging (EACVI) guidelines [19,20].
4. Electrocardiographic (ECG) Holter monitoring (24 h): Continuous ECG data were recorded to assess AF episodes, premature atrial and ventricular contractions, HRV indices, and other rhythm-related metrics. For ambulatory ECG Holter monitoring, we utilized the GE Seer 1000 and GE CardioMem 4000 Holter monitors (GE Healthcare, USA). HRV analysis was performed using GE CardioDay ECG Holter software, v2.6 (GE Healthcare, Chicago, IL, USA).
5. Plasma biomarker analysis: Blood samples were collected upon study enrollment, after at least 12 h of overnight fasting. Basic biochemical measurements, including brain natriuretic peptide (BNP) and high-sensitivity cardiac troponin I (hs-cTnI), were performed immediately after collection. Additional blood samples were obtained in vacuum collection tubes without heparin, EDTA, or gel (Becton, Dickinson and Company, Franklin Lakes, NJ, USA) and transferred to the laboratory within 1 h of vein puncture while maintained on ice. The samples were preserved on ice until centrifugation to minimize biochemical changes, as certain biomarkers, including Hcy, can continue to be produced by red blood cells post-collection. After a minimum of 45 min to allow clot formation, the blood samples were centrifuged at 4000 rpm for 10 min in a refrigerated centrifuge. The serum samples that were not analyzed immediately were stored at −80 °C. Lipemic or hemolyzed samples were excluded.

At the conclusion of this study, the stored serum samples were removed from the −80 °C freezer and allowed to thaw gradually in a refrigerated environment (2–8 °C) to maintain sample integrity. Once thawed, the samples were inspected for any signs of lipemia or hemolysis, and those that failed quality checks were excluded from further analysis. The remaining samples were centrifuged again at 4000 rpm for 10 min in a refrigerated centrifuge to ensure the removal of any residual particulate matter. Following this step, the supernatant was carefully collected and used for the analysis of Hcy and GAL3. Hcy and GAL3 were measured using the immunoassay analyzer Alinity-i (Abbott) by chemiluminescence (CMIA). These markers were chosen for their roles in inflammation, fibrosis, and myocardial stress, which are pivotal in AF pathophysiology.

#### 2.3.2. Follow-Up

The participants were followed for 12 months. During this period, comprehensive records were maintained for each patient to document the occurrence of new AF episodes. Recurrence was defined as any episode of AF lasting ≥30 s, in accordance with ESC Guidelines [4], and was identified through one of the following: (a) 24 h ECG Holter monitoring, (b) documentation in medical records, including arrhythmic events managed at external centers, or (c) patient-reported episodes consistent with prior confirmed AF, typically managed with self-administered antiarrhythmic drugs. Only objectively verified events based on ECGs, medical documentation, or consistent symptomatic episodes managed as AF were considered valid recurrence events.

#### 2.3.3. Outcomes

The primary outcome of this study was AF recurrence within 12 months of enrollment, while the time to first AF recurrence was identified as a secondary outcome. Even though the biomarkers were measured only at baseline, their prognostic significance for AF recurrence was evaluated.

### 2.4. Statistical Analysis

The statistical analysis included descriptive statistics to assess the quantitative and qualitative variables. For the quantitative variables, the mean value and the standard deviation were computed. For the qualitative variables, the frequency of the categories and the corresponding percentages were employed. The differences in each variable between the two AF states (recurrence/no recurrence) were assessed using either the independent samples t-test or the chi-squared test (as appropriate). Next, univariable binary logistic regression was employed to assess the impact of each variable of interest on the binary form of the variable recurrence after one year (1: at least one event in one year, 0: no events in one year). Particularly for GAL3, receiver operating characteristic (ROC) analysis was used to determine a binary threshold related to the recurrence after one year (using the Youden Index criterion), and, if appropriate, transform the quantitative GAL3 into a binary one. All variables that exhibited a *p*-value less than 0.05 in the univariable binary logistic regression analysis were then included in a multivariable binary logistic regression model. The estimated odds ratio (OR) for each independent quantitative variable reflected the ratio of the odds of recurrence when the independent variable increased by one unit to the odds of recurrence before this increase. The odds of recurrence were the ratio of the probability of recurrence occurring to the probability of it not occurring. For independent qualitative binary variables (Yes/No), the OR reflected the ratio of the odds with the “Yes” category to the odds with the “No” category (reference). For independent qualitative variables with more than two categories, the first category was used as the reference. Here, OR > 1 implied that increasing values of the predictor variable were related to an increasing probability of recurrence, while OR < 1 implied that increasing values of the predictor variable were related to a decreasing probability of recurrence.

Similarly, survival analysis was also conducted to evaluate the impact of each variable on the time that the recurrence occurred during the 1-year follow-up period. More specifically, the Cox regression model was employed. The estimated hazard ratio (HR) for each independent variable reflected the ratio of the hazards for recurrence when the independent variable increased by one unit (quantitative variables) or when each category was compared to the reference category (qualitative variables). Here, HR > 1 implied that increasing values of the predictor variable were related to a higher risk of recurrence, while HR < 1 implied that increasing values of the predictor variable were related to a lower risk of recurrence. The proportional hazards assumption for the Cox regression model was assessed by employing the “cox.zph” R function, which uses Schoenfeld residuals to test whether the effect of a covariate changes over time. The purpose of the Cox regression analysis was to assess whether the specific time that the recurrence occurred during the 1-year follow-up period (for example, early or late during this time interval) differentiated the results compared to the binary logistic regression.

The level of statistical significance was set at 0.05 in all cases, and that of marginal statistical significance was set at 0.10. All analyses were performed with IBM SPSS Statistics, version 22 (IBM Corp., Armonk, NY, USA), and the R programming language, version 4.4.1.

## 3. Results

### 3.1. Demographic and Lifestyle Characteristics

The baseline characteristics of the study population, stratified by AF recurrence during the 1-year follow-up (No/Yes), are presented in Table 1. The total cohort included 73 patients, with 42 patients in the no recurrence group and 31 in the recurrence group. The mean age of the cohort was 60 years (standard deviation [SD] 11.6), with no significant difference between the no recurrence and recurrence groups (*p* = 0.424). The sex distribution was similar between the two groups, with 47.9% male participants in the total cohort [42.9% in the no recurrence group vs. 54.8% in the recurrence group (*p* = 0.311)]. Smoking, alcohol consumption, and physical activity rates were comparable between the no recurrence and recurrence groups (*p* = 0.695, *p* = 0.542, and *p* = 0.368, respectively). These findings suggest no significant differences in lifestyle habits between the groups.

### 3.2. Atrial Fibrillation Characteristics

The total disease duration for AF was 40 months (SD 49.4), with no significant difference between groups (38.6 [SD 54.3] vs. 41.9 [SD 42.5], *p* = 0.781). Across both groups, the prevalence of PAF episodes in the last 6 months was comparable, with 81% of the participants in the no recurrence group and 77.4% in the recurrence group reporting episodes (*p* = 0.712). Similarly, the proportion of individuals experiencing PAF episodes in the last 1 month was almost identical, with 38.1% in the no recurrence group and 38.7% in the recurrence group (*p* = 0.957). These findings suggest a consistent burden of PAF episodes in our study cohort. Most of the patients reported symptoms (93.2% in the total cohort, 92.9% in the no recurrence group, and 93.5% in the recurrence group), with no significant difference between the groups (*p* = 0.908). The distribution of EHRA scores was similar between the groups: 23.3% of the total cohort had an EHRA score of 1, 65.8% had a score of 2, and 11.0% had a score of 3, with no significant differences between the groups (*p* = 0.540).

### 3.3. Comorbidities

Arterial hypertension was the most common comorbidity, present in 45.2% of the cohort, with no significant difference between the groups (*p* = 0.639). Obstructive sleep apnea (OSA) was more prevalent in the recurrence group (19.4% vs. 4.8%, *p* = 0.049). The prevalence of other comorbidities, including diabetes, dyslipidemia, coronary artery disease, stroke, valvular disease, and thyroid disease, did not differ significantly between the groups.

### 3.4. Echocardiographic Characteristics

The results regarding the echocardiographic parameters are displayed in Table 2. LVEF was similar between the no recurrence and recurrence groups (*p* = 0.126). There were no significant differences in left ventricular mass index (LVMI), GLS, or the other echocardiographic parameters, including E/E’, LA volume index (LAVI), and LA strain parameters. However, the RV FAC was significantly lower in the recurrence group (40.1% vs. 44.6%, *p* = 0.014), indicating worse right ventricular function.

### 3.5. CPET Results

Peak VO_2_ was comparable between the groups (18.3 vs. 19.0 mL/kg/min, *p* = 0.585), as were the other CPET parameters, including anaerobic threshold (VO_2_ AT), total work at VO_2_ max, and VO_2_/HR at VO_2_ max (Table 2). No significant differences were observed in VE/VCO_2_ or in partial pressure of end-tidal carbon dioxide (PETCO_2_), which remained similar across the groups (Table 2).

### 3.6. Twenty-Four-Hour ECG Holter Monitoring

Baseline 24 h ambulatory ECG Holter monitoring revealed a significantly higher incidence of AF episodes in the recurrence group at the time of recording (seven vs. zero episodes, *p* = 0.001), while no significant differences were demonstrated in the occurrence of other arrhythmias, such as atrial flutter. It is important to note that this finding reflects only the baseline Holter recordings and does not correspond to the total number of patients who experienced AF recurrence during the one-year follow-up (n = 31). The minimum 24 h heart rate was similar between the groups (*p* = 0.758), while the maximum heart rate was higher in the recurrence group (*p* = 0.031). Significant differences in HRV were also noted, with the recurrence group showing a higher SDNN (*p* = 0.078) and a higher SDRR (*p* = 0.015).

### 3.7. Regression

The univariable binary logistic regression (Table 3) revealed that three variables exhibited a statistically significant impact on the probability of recurrence after one year (1: at least one event in one year, 0: no events in one year). In particular, RV FAC was a positive factor with an odds ratio (OR) of 0.917 (*p* = 0.020), while the SDRR (OR = 1.053, *p* = 0.015) was a negative factor. Namely, increasing values of RV FAC were related to a decreasing probability of recurrence, while increasing values of the SDRR were related to an increasing probability of recurrence. On the other hand, GAL3 only marginally did not exhibit statistical significance (OR = 1.149, *p* = 0.051) when treated as a continuous variable. However, since GAL3 was of particular interest in this study as an established biomarker in AF, known for its potential role in fibrosis and remodeling processes, ROC analysis was employed to assess whether it would be appropriate to dichotomize GAL3. It was found that the area under the ROC curve was 0.663 (*p* = 0.019), indicating that the dichotomization of GAL3 would be beneficial. By employing the Youden Index criterion, the binary threshold was estimated to be 10.95, and GAL3 was transformed into a binary variable (1: GAL3 > 10.95, 0: GAL3 < 10.95). GAL3 > 10.95 ng/mL exhibited statistically significant negative impact on the probability of recurrence after one year with an OR of 6.127 (*p* = 0.001); namely, the odds of the patients with a GAL3 value higher than 10.95 were 6.127 times the odds of the patients with a GAL3 value less than 10.95. Of note, OSA exhibited a marginal statistically significant negative impact on the probability of recurrence after one year, with an OR of 4.800 (*p* = 0.067). Antiarrhythmic and beta-blocker use were also included in the univariate analysis and showed no significant association with AF recurrence. Specifically, the use of antiarrhythmic drugs was associated with an OR of 1.422 (95% CI: 0.560–3.614, *p* = 0.459), while the combination of beta-blockers and antiarrhythmic drugs showed an OR of 1.174 (95% CI: 0.455–3.025, *p* = 0.740).

After including RV FAC, SDRR, and GAL3 > 10.95 ng/mL in a multivariable binary logistic regression model (Table 4), it was found that SDRR and GAL3 > 10.95 ng/mL retained their statistical significance, with an OR of 1.061 (*p* = 0.021) and 5.206 (*p* = 0.006), respectively. On the other hand, RV FAC exhibited a marginal impact on recurrence, with an OR of 0.927 (*p* = 0.062).

The Cox regression analysis revealed similar results to the binary logistic regression. In particular, the only variables that exhibited statistical significance in the univariable model were RV FAC, SDRR, and GAL3 > 10.95 ng/mL. In addition to that, the estimates of HR were very similar in value to the corresponding estimates of the OR (HR: 0.948, *p* = 0.028; HR: 1.030, *p* = 0.003; and HR: 4.175, *p* = 0.002, respectively). By including all three predictors in a multivariable Cox regression model, it was found that SDRR and GAL3 > 10.95 ng/mL retained their statistical significance with an HR of 1.023 (*p* = 0.028, 95% CI-HR: 1.002–1.043) and 3.315 (*p* = 0.014, 95% CI-HR: 1.274–8.626), respectively. On the contrary, RV FAC was not statistically significant, with an HR of 0.976 (*p* = 0.365, 95% CI-HR: 0.927–1.028). The results of the proportional hazard assumption assessment for the multivariable Cox regression model showed that the assumption was not violated for any of the three variables (*p*: 0.36, 0.47, and 0.73, respectively).

## 4. Discussion

This pilot study emphasizes the feasibility of integrating CPET, echocardiography, and biomarkers for PAF recurrence prediction. The findings of this study underscore the utility of a multifaceted diagnostic approach, highlighting specific parameters such as right ventricular RV FAC, SDRR, and GAL3 levels as key predictors of AF recurrence. These insights have important clinical implications for improving risk stratification and personalizing management strategies in this challenging patient population.

Our analysis identified SDRR and GAL3 as independent predictors of PAF recurrence within one year. Higher SDRR and elevated GAL3 levels were significantly associated with increased recurrence risk, underlining the role of autonomic dysfunction and fibrosis in AF pathophysiology. Additionally, RV FAC demonstrated a trend toward significance, suggesting a possible association between RV function and AF recurrence. While not reaching full statistical significance, this finding aligns with previous studies that emphasize the importance of RV performance in maintaining sinus rhythm, suggesting that slight impairments in right ventricular performance may exacerbate atrial remodeling and electrical instability, thereby promoting AF recurrence. Incorporating RV FAC into routine echocardiographic assessments of patients with PAF may aid in the identification of high-risk individuals.

The consistency observed between the Cox regression and binary logistic regression models validates the robustness of our findings. Both models identified SDRR and GAL3 as statistically significant predictors, while RV FAC, although significant in the univariable analysis, did not retain significance in the multivariable Cox model. These results emphasize the importance of autonomic function and fibrotic pathways in predicting AF recurrence, aligning with prior studies that highlight the prognostic role of HRV and GAL3 levels in arrhythmia pathophysiology.

In the context of HRV analysis, SDRR, derived from 24 h ECG Holter monitoring, has been proven to correlate with SDNN, a commonly used measure of global HRV [21]. Studies have demonstrated that long-term recordings, such as those from 24 h ambulatory ECG Holter monitors, provide a robust and stable representation of autonomic nervous system activity, with SDRR serving as a valid indicator of overall HRV, even when NN intervals are not specifically isolated [22,23]. SDRR has been utilized as a comparable metric in assessing autonomic nervous system function, particularly when preprocessing for NN intervals is unavailable or impractical [22]. Although SDNN is more widely adopted, SDRR offers a practical and reliable alternative for analyzing variability in such datasets. In our study, higher SDRR values were linked with higher AF recurrence possibility, reflecting high sympathetic activity or impaired vagal tone, both of which are known contributors to AF pathogenesis. Although SDNN did not reach statistical significance in our study, the observed trend aligns with the existing literature, which consistently reports a correlation between increased SDNN values and higher AF recurrence risk [24].

GAL3 is a β-galactoside-binding protein that is proven to exacerbate fibrotic activity, whilst, at the same time, AF induces GAL3 production through tissue injury and structural and electrical remodeling [25]. This biomarker seems to play a pivotal role in cardiac fibrosis, as it is highly expressed in fibrotic tissues, such as the LA tissue of patients with AF, and it is upregulated in chronic inflammatory and fibrotic conditions [26,27]. GAL3 emerged as a robust biomarker in AF, as elevated levels have been strongly correlated with AF development, while GAL3 levels were found to be higher among patients with persistent AF, underlining the importance of this biomarker in the maintenance of the arrhythmia [28,29]. Our study is in accordance with the existing literature, identifying GAL3 as an independent and important predictor of AF recurrence. Targeting GAL3 through novel pharmacologic agents or lifestyle interventions may hold promise for modifying the AF trajectory.

OSA has been increasingly recognized as a significant comorbidity in patients with AF [30]. This condition is characterized by intermittent cessation of airflow during sleep, leading to repeated episodes of hypoxia and arousal; these events can trigger sympathetic activation, inflammation, and changes in autonomic tone, all of which can contribute to the development and recurrence of AF [31,32]. In our study, OSA exhibited a marginally significant negative impact on the probability of AF recurrence (OR 4.800, *p* = 0.067), suggesting that its presence may increase the risk of recurrence in patients with PAF. The association between OSA and AF recurrence has already been established, and several studies have shown that effective management, such as through continuous positive airway pressure (CPAP) therapy, may reduce AF burden and improve outcomes in affected patients [33,34,35]. The potential role of OSA in AF recurrence emphasizes the need for comprehensive risk stratification in patients with AF, which should include the evaluation of underlying conditions.

Hcy levels, along with BNP and hs-cTnI, did not demonstrate any significant association with AF relapse in our study. Despite the well-established roles of BNP and hs-cTnI as markers of cardiac stress and injury, and the recognized involvement of Hcy in vascular and thrombotic processes, none of these biomarkers appeared to provide additional prognostic value for predicting AF recurrence in the patient population studied [36,37,38]. This may reflect the relatively preserved cardiac function in our study population, reducing the sensitivity of these markers. Additionally, the dynamic nature of BNP in response to acute hemodynamic changes may limit its utility in stable outpatients. Future studies should explore whether combining BNP with markers of fibrosis, such as GAL3, could enhance predictive accuracy.

Antiarrhythmic drug therapy is a well-established determinant of AF recurrence and plays a central role in rhythm control strategies. In our study, although treatment with antiarrhythmic agents or their combination with beta-blockers was not a primary focus, we included these variables in the univariate regression analysis to account for potential confounding. Notably, neither antiarrhythmic drug use (OR: 1.422, 95% CI: 0.560–3.614, *p* = 0.459) nor the combined use of beta-blockers and antiarrhythmic therapy (OR: 1.174, 95% CI: 0.455–3.025, *p* = 0.740) was significantly associated with AF recurrence in our cohort. These findings may reflect the real-world heterogeneity in treatment choices and the fact that all patients were managed according to the ESC guidelines [4]. Nonetheless, the prognostic value of antiarrhythmic therapy warrants further investigation in larger, treatment-stratified cohorts.

Although CPET was central to the study design, its parameters, including peak VO_2_ and VE/VCO_2_, did not independently predict PAF recurrence. This finding contrasts with the existing literature that often highlights the utility of CPET in evaluating exercise tolerance and predicting cardiovascular outcomes in patients with AF and other cardiac conditions [8]. A possible explanation lies in the characteristics of our study population; the participants in the PLACEBO trial were relatively healthy individuals without significant comorbidities, as evidenced by preserved LVEF and the absence of structural cardiomyopathies. Previous studies have shown that the predictive value of CPET parameters is more pronounced in populations with advanced cardiac dysfunction or significant exercise intolerance; the apparent disconnect between CPET metrics and recurrence risk contrasts with some reports linking improved cardiorespiratory fitness to reduced AF burden [39,40]. This discrepancy may reflect differences in population characteristics or the interplay of exercise-induced atrial remodeling. Consequently, the limited variability in CPET metrics among our cohort may have diminished their prognostic utility. Furthermore, our findings align with reports suggesting that the role of CPET in AF prognosis may be more nuanced, dependent on the presence of additional comorbidities or the specific subtype of AF being studied [41]. While CPET remains a valuable tool for assessing overall cardiovascular health and exercise capacity, its application as an isolated prognostic marker for PAF recurrence warrants further investigation in diverse populations.

Our findings are consistent with previous studies that have examined individual components of the PLACEBO model. For instance, the prognostic value of right ventricular function and GAL3 has been validated in broader AF cohorts [42,43,44]. However, the combination of these markers with HRV metrics represents a novel approach that integrates the individual strengths of these diverse diagnostic modalities. In the same line, even though CPET variables such as peak VO_2_ and VE/VCO_2_ were not independently predictive in our cohort, they provide critical context for interpreting overall cardiovascular health and may enhance the predictive capacity of other markers in upcoming studies. Future studies with larger multicenter cohorts and extended follow-up periods are needed to validate these preliminary findings and refine the proposed diagnostic approach.

### Study Limitations

The most important limitation of this study is that, by design, it is a pilot study; therefore, the results should be interpreted with caution. The sample size, although relatively small, is appropriate for an exploratory analysis. Additionally, the follow-up period of 12 months may not fully capture the long-term recurrence patterns of AF; some patients may have developed recurrence beyond the study period, potentially influencing the interpretation of the predictive markers. Furthermore, while all patients underwent 24 h ECG Holter monitoring and recurrence was documented based on medical records or verified symptomatic episodes, continuous rhythm monitoring was not performed, which may have led to the underdetection of asymptomatic episodes. Furthermore, adherence to antiarrhythmic therapy during follow-up was not formally verified, which may have affected recurrence patterns and represents a further limitation of our analysis. Despite these limitations, our study serves as an important first step in evaluating the feasibility of a multimodal approach to predicting AF recurrence. The exploratory insights that have emerged should be further assessed and confirmed in larger and potentially multicenter studies. Longer follow-up durations may contribute to more effectively assessing the chronicity of AF recurrence and its impact on clinical outcomes.

## 5. Conclusions

The PLACEBO pilot trial demonstrates the feasibility and clinical relevance of a multimodal diagnostic approach for predicting PAF recurrence. Our findings establish GAL3 and SDRR as significant predictors, while RV FAC demonstrates a potential association with recurrence. These results emphasize the value of integrating functional, structural, and biochemical assessments in routine practice for more comprehensive risk stratification. Future research should focus on validating these findings in larger, more diverse populations and investigating their potential role in guiding personalized therapeutic strategies.

## Figures and Tables

**Table 1:** Baseline characteristics are displayed for the total cases and, separately, within the two categories of the variable representing recurrence in 1 year (No/Yes). For quantitative variables, the mean value and the standard deviation (in parentheses) are displayed. For qualitative variables, the frequency of the stated category (in parentheses) is presented along with the corresponding percentage (in parentheses). All data refer to the total study population, including both male and female participants.

Variables	Total Cases (n = 73)	AF Recurrence During 1 y Follow-Up NO (n = 42)	AF Recurrence During 1 y Follow-Up YES (n = 31)	*p*-Values
Age (years)	60.0 (11.6) Range: 18.3–80.1	59.1 (13.1) Range: 18.3–75.9	61.3 (9.2) Range: 34.8–80.1	0.424 ^a^
Male sex (n, %)	35 (47.9%)	18 (42.9%)	17 (54.8%)	0.311 ^b^
Female sex (n, %)	38 (52.1%)	24 (57.1%)	14 (45.2%)	0.311 ^b^
BMI (kg/m^2^)	27.9 (4.4)	28.2 (4.6)	27.4 (4.1)	0.414 ^a^
BSA (m^2^)	2.0 (0.2)	1.9 (0.2)	1.9 (0.2)	0.695 ^a^
Smoking (n, %)	23 (31.5%)	14 (33.3%)	9 (29.0%)	0.695 ^b^
Alcohol (n, %)	37 (50.7%)	20 (47.6%)	17 (54.8%)	0.542 ^b^
Physical activity (n, %)	50 (68.5%)	27 (64.3%)	23 (74.2%)	0.368 ^b^
**AF characteristics**				
Total disease duration (months)	40.0 (49.4)	38.6 (54.3)	41.9 (42.5)	0.781 ^a^
PAF episode in the last 6 months (n, %)	58 (79.5%)	34 (81%)	24 (77.4%)	0.712 ^b^
PAF episode in the last 1 month (n, %)	28 (38.4%)	16 (38.1%)	12 (38.7%)	0.957 ^b^
Presence of symptoms (n, %)	68 (93.2%)	39 (92.9%)	29 (93.5%)	0.908 ^b^
EHRA score				0.540 ^b^
1 (n, %)	17 (23.3%)	10 (23.8%)	7 (22.6%)	
2 (n, %)	48 (65.8%)	26 (61.9%)	22 (71.0%)	
3 (n, %)	8 (11.0%)	6 (14.3%)	2 (6.5%)	
**Comorbidities**				
Arterial hypertension (n, %)	33 (45.2%)	18 (42.9%)	15 (48.4%)	0.639 ^b^
Diabetes mellitus (n, %)	6 (8.2%)	3 (7.1%)	3 (9.7%)	0.697 ^b^
Dyslipidemia (n, %)	36 (49.3%)	20 (47.6%)	16 (51.6%)	0.736 ^b^
OSA (n, %)	8 (11.0%)	2 (4.8%)	6 (19.4%)	0.049 ^b^
Coronary artery disease (n, %)	3 (4.1%)	2 (4.8%)	1 (3.2%)	0.744 ^b^
Stroke (n, %)	2 (2.7%)	0 (0.0%)	2 (6.5%)	0.095
Valvular disease (n, %)	3 (4.1%)	3 (7.1%)	0 (0.0%)	0.129 ^b^
Thyroid disease (n, %)	17 (23.3%)	11 (26.2%)	6 (19.4%)	0.495 ^b^

^a^: Comparison based on the independent samples t-test. ^b^: Comparison based on the chi-squared test. Abbreviations: BMI, body mass index; BSA, body surface area; AF, atrial fibrillation; PAF, paroxysmal atrial fibrillation; EHRA, European Heart Rhythm Association; OSA, obstructive sleep apnea.

**Table 2:** Echocardiographic, CPET, and 24 h ECG Holter monitoring values and biomarker measurements are displayed for the total cases and, separately, within the two categories of the variable representing recurrence in 1 year (No/Yes). For quantitative variables, the mean value and the standard deviation (in parentheses) are displayed. For qualitative variables, the frequency of the stated category (in parentheses) is presented along with the corresponding percentage (in parentheses).

Variables	Total Cases (n = 73)	AF Recurrence Within 1 y Follow-Up NO (n = 42)	AF Recurrence Within 1 y Follow-Up YES (n = 31)	*p*-Values
**Echocardiography**				
LVEF (%)	58.8 (13.6)	57.0 (17.1)	61.4 (5.4)	0.126 ^a^
LVMI (g/m^2^)	92.4 (24.6)	90.4 (19.3)	95.1 (30.4)	0.431 ^a^
GLS (%)	−18.3 (2.7)	−18.6 (2.4)	−17.9 (3.1)	0.339 ^a^
E/E’	8.4 (4.1)	8.7 (4.9)	7.9 (2.9)	0.441 ^a^
LAVI (mL/m^2^)	30.2 (9.9)	31.1 (9.7)	29.0 (10.1)	0.374 ^a^
LAVpreA (mL)	43.6 (17.2)	44.8 (17.7)	42.0 (16.8)	0.509 ^a^
LA strain reservoir (%)	24.8 (7.6)	24.0 (7.4)	26.0 (8.0)	0.275 ^a^
LA strain conduit (%)	−14.3 (5.0)	−14.4 (5.0)	−14.2 (5.0)	0.833 ^a^
LA strain contraction (%)	−11.5 (4.4)	−11.0 (4.5)	−12.1 (4.2)	0.266 ^a^
RV FAC (%)	42.7 (7.9)	44.6 (8.5)	40.1 (6.3)	0.014 ^a^
RV FWS (%)	−23.0 (6.0)	−24.5 (6.1)	−21.3 (5.4)	0.053 ^a^
RV GS (%)	−20.6 (4.4)	−21.7 (4.5)	−19.3 (4.1)	0.051 ^a^
TAPSE (cm)	2.6 (0.4)	2.6 (0.5)	2.5 (0.4)	0.401 ^a^
**CPET**				
Peak VO_2_ (mL/kg/min)	18.6 (5.2)	18.3 (5.6)	19.0 (4.8)	0.585 ^a^
VO_2_ AT (mL/kg/min)	12.7 (3.5)	12.7 (3.8)	12.8 (3.2)	0.863 ^a^
Work at VO_2_ max (Watts)	108.2 (55.4)	102.4 (59.0)	116.2 (50.0)	0.296 ^a^
VO_2_/HR at VO_2_ max (mL/beat)	11.8 (3.4)	11.8 (3.0)	12.0 (3.9)	0.804 ^a^
VE/VCO_2_ at VO_2_ max	30.4 (4.7)	30.4 (5.4)	30.5 (3.7)	0.967 ^a^
PETCO_2_ at VO_2_ max (mmHg)	36.0 (12.0)	35.4 (13.7)	36.9 (9.4)	0.606 ^a^
**24 h ECG Holter monitoring**				
Total PVCs in 24 h	46.3 (124.1)	41.5 (127.5)	53.0 (121.0)	0.707 ^a^
Total PACs in 24 h	804.5 (1866.5)	620.4 (1583.8)	1067.7 (2213.4)	0.334 ^a^
Atrial fibrillation (n)	7 (10.1%)	0 (0.0%)	7 (24.1%)	0.001 ^b^
Atrial flutter (n)	1 (1.4%)	0 (0.0%)	1 (3.4%)	0.237 ^b^
Minimum heart rate (bpm)	51.1 (6.4)	51.0 (4.8)	51.4 (8.1)	0.758 ^a^
Maximum heart rate (bpm)	112.2 (20.1)	107.8 (16.6)	118.3 (23.0)	0.031 ^a^
QTc (ms)	443.8 (23.1)	444.0 (21.0)	443.4 (26.0)	0.920 ^a^
SDNN (ms)	144.6 (38.8)	137.6 (37.6)	154.2 (39.1)	0.078 ^a^
SDANN (ms)	122 (37.1)	117.7 (34.4)	127.9 (40.4)	0.264 ^a^
SDRR (ms)	39.4 (14.9)	35.4 (10.5)	45.0 (18.2)	0.015 ^a^
HRV-TI	30.6 (8.9)	30.0 (8.6)	32.1 (9.3)	0.258 ^a^
PNN50 (%)	12.8 (14.2)	11.3 (13.9)	15.0 (15.0)	0.301 ^a^
RMSSD (ms)	70.1 (55.1)	62.2 (45.6)	81.0 (65.4)	0.190 ^a^
SDSD (ms)	60.1 (48.4)	53.0 (40.0)	70.1 (57.2)	0.143 ^a^
Deceleration capacity (ms)	5.8 (2.8)	5.7 (2.3)	5.9 (3.4)	0.733 ^a^
Acceleration capacity (ms)	−7.9 (3.5)	−7.4 (2.7)	−8.6 (4.3)	0.140 ^a^
**Biomarkers**				
hs-cTnI (pg/mL)	6.6 (18.4)	8.1 (24.0)	4.5 (4.2)	0.413 ^a^
BNP (pg/mL)	89.1 (75.6)	95.7 (81.1)	79.8 (67.5)	0.381 ^a^
Hcy (μmol/L)	12.3 (3.9)	12.5 (4.2)	12.1 (3.5)	0.692 ^a^
GAL3 (ng/mL)	12.2 (3.6)	11.4 (3.7)	13.2 (3.4)	0.045 ^a^

^a^: Comparison based on the independent samples t-test. ^b^: Comparison based on the chi-squared test. Abbreviations: LVEF, left ventricular ejection fraction; LVMI, left ventricular mass index; GLS, global longitudinal strain; E/E’, ratio of early diastolic transmitral flow velocity to early diastolic mitral annular velocity; LAVI, left atrial volume index; LA VpreA, left atrial volume before atrial contraction; RV FAC, right ventricular fractional area change; RV FWS, right ventricular free wall strain; RV GS, right ventricular global strain; TAPSE, tricuspid annular plane systolic excursion; CPET, cardiopulmonary exercise testing; peak VO_2_/VO_2_ max, peak oxygen consumption; VO_2_ AT, oxygen consumption at anaerobic threshold; VO_2_/HR, oxygen consumption per heart rate; VE/VCO_2_, ventilatory equivalent for carbon dioxide; PETCO_2_, partial pressure of end-tidal carbon dioxide; PVCs, premature ventricular contractions; PACs, premature atrial contractions; bpm, beats per minute; QTc, corrected QT interval; SDNN, standard deviation of normal-to-normal R-R intervals; SDANN, standard deviation of the average NN intervals; SDRR, standard deviation of RR intervals; HRV-TI, heart rate variability triangular index; PNN50, percentage of differences between adjacent NN intervals greater than 50 ms; RMSSD, root mean square of successive differences between adjacent NN intervals; SDSD, standard deviation of successive differences; BNP, brain natriuretic peptide; hs-cTnI, high-sensitivity cardiac troponin I; Hcy, homocysteine; GAL3, galectin-3.

**Table 3:** The results of the univariable binary logistic regression are displayed for all the variables considered. The dependent variable in the analysis is AF recurrence within 1 year of follow-up. The odds ratio (OR) for each independent quantitative variable reflects the ratio of the odds of recurrence when the independent variable increases by one unit to the odds of recurrence before this increase (the odds of recurrence are the ratio of the probability of recurrence occurring to the probability of it not occurring). For independent qualitative variables, the OR reflects the ratio of the odds of each category to the odds of the reference category (as appropriately denoted). An estimated OR > 1 implies that increasing values of the predictor variable are related to an increasing probability of recurrence, while an OR < 1 implies that increasing values of the predictor variable are related to a decreasing probability of recurrence.

			95% Conf. Interval—OR	
Variable	Odds Ratio (OR)	*p*-Value	Lower	Upper
Sex—male	0.618	0.312	0.243	1.573
Age (years)	1.017	0.420	0.976	1.061
Weight (kg)	1.000	0.983	0.968	1.032
Height (cm)	1.031	0.270	0.977	1.087
BMI (kg/m^2^)	0.955	0.410	0.857	1.065
BSA (m^2^)	1.623	0.690	0.150	17.516
Total disease duration (months)	1.001	0.778	0.992	1.011
Total PAF episodes (n)	1.120	0.330	0.892	1.406
PAF episodes in the last 6 months—Yes	1.040	0.865	0.660	1.640
PAF episodes in the last 1 month—Yes	1.267	0.585	0.542	2.963
Presence of symptoms—Yes	1.115	0.908	0.175	7.112
EHRA Score 2 vs. 1 3 vs. 1	1.209 0.476	0.740 0.437	0.394 0.073	3.706 3.087
Thyroid disease—Yes	0.676	0.496	0.219	2.085
OSA—Yes	4.800	0.067	0.898	25.665
Smoking—Yes	0.818	0.696	0.299	2.239
Diabetes mellitus—Yes	1.393	0.698	0.262	7.416
Arterial hypertension—Yes	1.250	0.639	0.492	3.176
Dyslipidemia—Yes	1.173	0.736	0.463	2.971
Alcohol—Yes	1.336	0.542	0.526	3.389
Physical activity—Yes	1.597	0.369	0.574	4.441
Use of beta-blocker—Yes	0.692	0.453	0.265	1.807
Use of antiarrhythmic drugs—Yes	1.422	0.459	0.560	3.614
Use of beta-blocker + antiarrhythmic drugs—Yes	1.174	0.740	0.455	3.025
WBC (cells/mL)	1.000	0.188	1.000	1.001
Hb (g/dL)	0.964	0.820	0.705	1.318
Ht (%)	1.016	0.840	0.872	1.184
RDW (%)	1.036	0.878	0.656	1.637
PLT (platelets/mL)	1.004	0.401	0.995	1.012
Mg (mg/dL)	0.333	0.361	0.031	3.525
Fe (μg/dL)	0.989	0.196	0.972	1.006
UIBC (μg/dL)	0.998	0.751	0.987	1.010
CRP (mg/L)	0.997	0.969	0.847	1.173
Ferritine (ng/mL)	0.998	0.565	0.992	1.005
hs-cTnI (ng/mL)	0.984	0.468	0.944	1.027
BNP (pg/mL)	0.997	0.378	0.991	1.004
PCT × 1000 (ng/mL × 1000)	1.011	0.658	0.962	1.063
Hcy (μmol/L)	0.975	0.687	0.862	1.103
GAL3 (ng/mL)	1.149	0.051	0.999	1.322
GAL3 > 10.95 ng/mL	6.127	0.001	2.074	18.103
Work at AT (Watts)	1.004	0.590	0.989	1.019
Work at VO_2_ max (Watts)	1.005	0.292	0.996	1.013
VT at VO_2_ max (L)	1.830	0.144	0.814	4.115
VO_2_ AT (mL/kg/min)	1.012	0.861	0.887	1.155
VO_2_ max (mL/kg/min)	1.026	0.580	0.938	1.121
VO_2_/HR at AT (mL/beat)	1.002	0.984	0.842	1.191
VO_2_/HR at VO_2_ max (mL/beat)	1.019	0.800	0.880	1.181
VE/VCO_2_ at AT *	1.017	0.770	0.906	1.142
VE/VCO_2_ at VO_2_ max *	1.002	0.966	0.908	1.106
PETCO_2_ at AT (mmHg)	1.008	0.685	0.971	1.046
PETCO_2_ at VO_2_ max (mmHg)	1.010	0.621	0.971	1.051
GLS (%)	1.105	0.334	0.903	1.352
LV mass (gr)	1.004	0.406	0.995	1.012
LVMI (g/m^2^)	1.008	0.430	0.988	1.028
LA diameter (cm)	1.042	0.919	0.470	2.310
LA volume max (mL)	0.991	0.467	0.967	1.015
LA volume min (mL)	0.985	0.397	0.950	1.021
LAVI (mL/m^2^)	0.978	0.370	0.931	1.027
LA ejection fraction (%)	1.024	0.352	0.974	1.077
LAVpreA (mL)	0.990	0.505	0.963	1.019
LA strain reservoir (%)	1.036	0.273	0.973	1.103
LA strain conduit (%)	1.010	0.830	0.920	1.110
LA strain contraction (%)	0.940	0.263	0.843	1.048
RV FAC (%)	0.917	0.020	0.853	0.986
RV FWS (%)	1.108	0.060	0.996	1.232
RV GS (%)	1.150	0.059	0.995	1.330
Total PVCs in 24 h (n)	1.001	0.704	0.997	1.005
Total SVE in 24 h (n)	1.000	0.346	1.000	1.000
QTc (msec)	0.999	0.918	0.978	1.020
SDNN (ms)	1.012	0.082	0.999	1.025
SDANN (ms)	1.008	0.262	0.994	1.021
SDRR (ms)	1.053	0.015	1.010	1.098
HRVTI *	1.033	0.255	0.977	1.091
PNN50 (%)	1.018	0.303	0.984	1.054
RMSSD (ms)	1.006	0.171	0.997	1.015
SDNN index	1.014	0.131	0.996	1.033
SDSD (ms)	1.008	0.152	0.997	1.018
Deceleration capacity (ms)	1.032	0.729	0.865	1.231
Acceleration capacity (ms)	0.888	0.177	0.748	1.055

Variables marked with * are dimensionless. Abbreviations: BMI, body mass index; BSA, body surface area; PAF, paroxysmal atrial fibrillation; EHRA, European Heart Rhythm Association; OSA, obstructive sleep apnea; WBC, white blood cell count; Hb, hemoglobin; Ht, hematocrit; RDW, red cell distribution width; PLT, platelet count; Mg, magnesium; Fe, iron; UIBC, unsaturated iron-binding capacity; CRP, c-reactive protein; hs-cTnI, high-sensitivity cardiac troponin I; BNP, brain natriuretic peptide; PCT, procalcitonin; Hcy, homocysteine; GAL3, galectin-3; AT, anaerobic threshold; VO_2_, oxygen uptake; HR, heart rate; VE/VCO_2_, ventilatory equivalent for carbon dioxide; PETCO_2_, end-tidal partial pressure of carbon dioxide; GLS, global longitudinal strain; LV, left ventricle; LVMI, left ventricular mass index; LA, left atrium; LAVI, left atrial volume index; RV, right ventricle; FAC, fractional area change; FWS, free wall strain; GS, global strain; PVCs, premature ventricular contractions; SVE, supraventricular ectopics; QTc, corrected qt interval; SDNN, standard deviation of normal to normal R-R intervals; SDANN, standard deviation of the average normal to normal R-R intervals in 5-minute segments over a 24 h period; HRVTI, heart rate variability triangular index; PNN50, percentage of successive nn intervals >50 ms; RMSSD, root mean square of successive differences; SDSD, standard deviation of successive differences.

**Table 4:** Multivariable binary logistic regression analysis for AF recurrence. The dependent variable in the model is AF recurrence within 1 year of follow-up. The odds ratio (OR) for each independent quantitative variable reflects the ratio of the odds of recurrence when the independent variable increases by one unit to the odds of recurrence before this increase (the odds of recurrence are the ratio of the probability of recurrence occurring to the probability of it not occurring). For independent qualitative variables, the OR reflects the ratio of the odds of each category to the odds of the reference category (as appropriately denoted). An estimated OR > 1 implies that increasing values of the predictor variable are related to an increasing probability of recurrence, while an OR < 1 implies that increasing values of the predictor variable are related to a decreasing probability of recurrence.

			95% Conf. Interval—OR	
Variable	Odds Ratio (OR)	*p*-Value	Lower	Upper
GAL3 > 10.95 ng/mL	5.206	0.006	1.618	16.748
RV FAC (%)	0.927	0.062	0.856	1.004
SDRR (ms)	1.061	0.021	1.009	1.116

Abbreviations: GAL3, galectin-3; RV FAC, right ventricular fractional area change; SDRR, standard deviation of RR intervals.

## Data Availability

Data are available on request due to restrictions (personal medical data).

## Abbreviations

The following abbreviations are used in this manuscript:

AF	atrial fibrillation
PAF	paroxysmal atrial fibrillation
CPET	cardiopulmonary exercise testing
TTE	transthoracic echocardiography
RV FAC	right ventricular fractional area change
LA	left atrial
HCM	hypertrophic cardiomyopathy
GAL3	galectin-3
Hcy	homocysteine
HRV	heart rate variability
BMI	body mass index
Peak VO_2_	peak oxygen uptake
VE/VCO_2_	minute ventilation/carbon dioxide production ratio
LV	left ventricular
LVEF	left ventricular ejection fraction
GLS	global longitudinal strain
EACVI	European Association of Cardiovascular Imaging
ECG	electrocardiogram
BNP	brain natriuretic peptide
Hs-cTnI	high-sensitivity cardiac troponin I
CMIA	chemiluminescence
ROC	receiver operating characteristics
SD	standard deviation
OSA	obstructive sleep apnea
LVMI	left ventricular mass index
LAVI	left atrial volume index
VO_2_ AT	anaerobic threshold
PETCO_2_	partial pressure of end-tidal carbon dioxide
OR	odds ratio
SDNN	standard deviation of normal to normal R-R intervals
SDRR	standard deviation of R-R intervals
